# Supplementary material for: Inactivation of NMDAR and CaMKII signaling within the prelimbic cortex blocks incubated cocaine- and sucrose-craving
Source: Neuropsychopharmacology. 2026 Jan 8;51(7):1197–206. doi: 10.1038/s41386-025-02310-0 (PMC13213044; doi:10.1038/s41386-025-02310-0)
Supplement: Supplementary file 1 — Supplemental Material [file 41386_2025_2310_MOESM1_ESM.docx]

**SUPPLEMENTAL MATERIALS**

**Methods**

*Subjects.* Adult male (225-250 g) and female Sprague Dawley rats (200-225 g; Charles River Laboratories, Hollister, CA, USA), were housed under a reverse light-dark cycle (lights off:10:00 am), with *ad libitum* access to food and water. The rats employed for immunoblotting were the same as those described in recent reports for incubated cocaine- [1] and sucrose-craving [2] and thus, no new rats were required to obtain the immunoblotting data described herein.

*Surgery.* Rats employed in the neuropharmacological studies of incubated cocaine-craving that were slated for testing in early withdrawal (WD1) underwent both intracranial (IC) and intravenous (IV) surgical procedures on the same day during a single surgical session, while rats slated for testing in late withdrawal (withdrawal day 30 or later; WD30+) underwent IV catheterization prior to cocaine self-administration procedures and IC surgery 7 days prior to their test for incubated craving (conducted on WD30 or later) to minimize subject attrition due to cannulae occlusion. Rats employed in the immunoblotting study of incubated cocaine-craving underwent IV surgical procedures only.  All surgical procedures were performed under isoflurane anesthesia (4% induction, 1-3% maintenance; Covetrus, Portland, ME). For IV catheterization, a chronic polyurethane catheter (12 cm long; 0.023 inner diameter, 0.038 in outer diameter; Instech Laboratories, Plymouth Meeting, PA) was inserted into the right jugular vein and tunneled subcutaneously along the shoulder to a back inclusion where it was connected to a threaded, protruding 22-gauge metal guide cannula (P1 Technologies, Roanoke, VA), within a rat infusion harness (Instech Laboratories, Plymouth Meeting, PA). The cannula was capped with both a plastic and a metal cap to prevent infection and immediately flushed with 0.1 ml of sterile cefazolin (100 mg/ml) and 0.1 ml of sterile heparin (70 U/ml).  Bilateral cannulae were implanted above the PL (AP: +3.0; ML: ±0.75, DV: -2.00 mm from Bregma) and secured to the skull with dental acrylic. Note that rats slated for testing in early withdrawal underwent the bilateral cannulation surgery immediately following IV catheterization and 7 days of post-operative care.  To minimize subject attrition due to clogged guide cannulae, rats slated for testing in later withdrawal underwent intracranial cannulation 7 days prior to cue testing, followed by 4 days of post-operative care.  For 48 hours post-procedure, rats were injected subcutaneously with Meloxicam (2 mg/kg) once a day to alleviate pain and inflammation. For rats slated for cocaine self-administration, post-operative care also consisted of daily IV infusions of cefazolin and heparin to maintain catheter patency. Catheter patency was confirmed just before cocaine self-administration training by administration of 0.1 ml of sodium Brevital (10 mg/ml) IV by loss of muscle tone.

*Cocaine and Sucrose Reinforcement.* Rats were randomly assigned to groups that were trained to self-administer IV cocaine (5-second IV infusion of 0.25mg/kg/0.1ml; MilliporeSigma, Burlington, MA) or 45 mg banana-flavored sucrose pellets (BioServ, Flemington, NJ) for 6hr/day over 10 days under an FR1 schedule of reinforcement with a 20-sec time-out. In both cases, an active lever-press resulted in reinforcer delivery and a 20-second tone-light stimulus complex (78 dB, 2 kHz) signaling that delivery. Any active lever presses that occurred during the 20-second stimulus delivery were recorded, but had no programmed consequences. To prevent potential overdose in cocaine trials, rats were limited to a maximum of 100 infusions for the first day. For the immunoblotting studies detailed below, a subset of rats were employed as controls to determine baseline protein expression in the cocaine/sucrose-naïve state. These Control rats were placed into their respective operant chambers for 6 h/day, but responding on neither the active nor the inactive levers resulted in any programmed consequences. After all rats completed 10 days of self-administration or control procedures, they were slated to undergo testing in either early withdrawal (WD1) or following at least 30 days of withdrawal (WD30+). One exception to the WD1 early withdrawal time-point occurred in our study of the protein correlates of incubated cocaine-seeking. In these studies, tissue was collected on WD3 to be consistent with the early withdrawal time-point employed in our earlier immunoblotting studies of the entire ventromedial PFC [e.g., 1,3,4] in order to facilitate direct comparisons across the present and prior results. Assignment to the testing groups was pseudo-random, ensuring comparable behavior during the operant-conditioning phase of each study across the different experimental conditions. During the periods of withdrawal, rats remained housed in the colony room and continued to receive *ad libitum* access to food and water. Any rats emitting less than ten active lever presses in the last three days of self-administration training were dropped from the study (did not undergo withdrawal or testing) and not included in the final statistical analyses of the results.

*Immunoblotting.* Immediately following the end of a 2-h Cue Test, brains were extracted and sectioned along the coronal plane (1 mm thick) using a rat brain mold (Braintree Scientific, Braintree, MA). The PL and IL subregions of the mPFC were then dissected over ice. For this, angled incisions were made using thin forceps medial to the anterior corpus callosum to separate grey from white matter. The resulting triangular piece of grey matter tissue was then segmented as follows: the portion of the tissue that lay dorsal to the genu of the corpus callosum was considered anterior cingulate cortex (which was not examined herein), the portion of tissue running parallel with the genu of the corpus callosum labeled as the PL, the tissue that ran parallel with the rostrum of the corpus callosum was considered as IL and any remaining tissue ventral to the IL dissection was discarded (see Figure 1 for a depiction of the PL vs. IL dissection sites). The same experimenter conducted all tissue dissection to ensure consistency both within and across experiments. PL and IL tissue was homogenized in a lysis buffer solution consisting of 10 mL RIPA Buffer (Boston Bioproducts, Milford, MA), 0.2 M sodium orthovanadate, 0.144 M sodium fluoride, 100 µL Phosphatase Inhibitor Cocktail 3 (MilliporeSigma, Burlington, MA), and a complete Mini-Tab Protease Inhibitor Cocktail Tablet (Roche Diagnostics, Mannheim, Germany). Homogenates were then centrifuged at 10,000 RPM for 20 min and the supernatant of the homogenates were stored at 80°C. Protein samples (19 𝜇l/lane) were subjected to SDS-PAGE on Tris-acetate gradient gels (3–8%) (Invitrogen), followed by wet polyvinylidene difluoride (MilliporeSigma, Burlington, MA) membrane transfer and membranes were preblocked with TBS containing 0.1% (v/v) Tween 20 and 5% (w/v) nonfat dried milk powder or BSA for a minimum of 1 h before overnight incubation with primary antibody. The following rabbit antibodies were used: GluN1 (1:250 dilution; Cell Signaling Technology; 5704S), GluN2A (1:250 dilution; MilliporeSigma; 07-632), p(Thr286)-CaMKII (1:1000 dilution; Cell Signaling Technology; 3361); GluN3A (1:150 dilution; Antibodies Incorporated; N416/40); GluN3A (1:150 dilution; MilliporeSigma; 07-356). The following mouse antibodies were also used: GluN2B (1:1000 dilution; Invitrogen; MA1-2014) and  CaMKII (1:1000 dilution; Millipore; 05-532). Calnexin was used to control for protein loading and transfer (1:1000 dilution; Enzo Life Sciences; ADI-SPA-860). Membranes were washed with TBST, rinsed with TBS, and incubated in either a goat anti-rabbit IRDye 800CW secondary antibody (1:10,000 dilution; Li-Cor; 925-3221) or a goat anti-mouse IRDye 680RD secondary antibody (1:10,000 dilution; Li-Cor; 925-68070) and imaged in an Odyssey Fc Infrared Imaging System (Li-Cor Biosciences, Lincoln, NE, USA). Protein expression was then quantified using Image Studio, raw values were normalized to the corresponding Calnexin signal, and then averaged to their control groups (WD3-Control for the cocaine study; WD1-Male for the sucrose study). As neither GluN3A antibody reliably detected this subunit in our tissue, the data for GluN3A not presented.

*Vaginal Cytology.* Evidence suggests that the magnitude of incubated cocaine-craving varies as a function of the estrus cycle [5-7]. Thus, we monitored each cocaine-experienced female's estrous cycle via vaginal swabbing following each cue test. Vaginal samples were collected by gently swabbing the vaginal canal with a cotton-tipped applicator, soaked in sterile saline and then smeared onto glass microscope slides, sprayed with a fixative, and stained with giemsa. Estrous stage was determined based on the presence and morphology of cells as in our recent study [1].

*Histological Verification of Microinjector Placement*. In the neuropharmacological studies, rats were deeply anesthetized under 4% isoflurane, brains extracted and fixed in 4% PFA solution, followed by a 20% sucrose solution for cryoprotection. Brains were then sectioned (30 μm thick) using a cryostat and slides were stained using Cresyl Violet. Microinjector placements were determined under a light microscope. Consistent with the areas of dissection employed in the immunoblotting experiments (see above), microinjector tips that were bilaterally located within the gray matter that ran parallel to the genu of the corpus callosum were deemed to be correctly localized to the PL (see Figures 2 and 3 for exact location of microinjector tips within the PL). Microinjector tips that were more dorsal to the genu were considered to be in the anterior cingulate cortex, while those that were more ventral to the genu and ran in parallel with the rostrum of the corpus callosum were considered to be in the IL. The hit rate for PL placement within each cohort of rats employed in the neuropharmacological studies was approximately 90% (e.g., 27/30) rats in each cohort). As there was an insufficient number of rats within each intracranial treatment group with microinjectors located outside the PL to examine how misplacement influenced the treatment effect, only rats whose microinjector tips were located in the PFC tissue parallel to the genu of the corpus callosum were included in the data analyses.

*Statistical Analyses*.  Consistent with our recent study of AMPA receptor correlates of incubated cocaine-craving [1], the immunoblotting data were normalized to the average of the two or three cocaine-naive Control-WD3 animals on each membrane and analyzed using a Group (Control vs. Cocaine) X Withdrawal (WD3 vs. WD30) ANOVA, separately for male and female subjects. The immunoblotting study of incubated sucrose-craving did not include a sucrose-naive control [2]. Thus, the samples from males and females were immunoblotted concurrently, the data normalized to the average of the three Male WD1 rats on each gel and then analyzed using a Sex X Withdrawal (WD1 vs. WD30) ANOVA. To relate protein expression to estrous phase, the immunoblotting data were analyzed using a Phase (estrus, diestrus, proestrus; no rats were found to be in metestrus) X Withdrawal ANOVA [1]. For the neuropharmacological studies of incubated craving, the average number of active and inactive lever presses emitted during either Cue Test was analyzed using a Treatment (WD1-VEH, WD30+-VEH, WD30+-AP5, WD30+-myr-AIP, WD30+-AP5+myr-AIP) X Sex ANOVA. For the neuropharmacological experiment conducted on WD1, the data were analyzed using a Treatment (VEH vs. drug)  X Sex ANOVA, separately for D-AP5 and myr-AIP as these drugs were examined in distinct experiments. Significant main effects or interactions were further investigated with t-tests using Bonferroni corrections for multiple comparisons. As the results of the statistical analyses of the neuropharmacological data failed to indicate any main Sex effects or interactions (p>.999), the data were collapsed across male and female subjects in all such experiments. Outliers were identified and excluded from the analyses using the ± 1 × IQR rule; however, in instances where too many outliers were identified, we adopted the ± 3 × IQR rule to ensure that only the most extreme outliers were removed. Alpha was set to 0.05. IBM SPSS Statistics software (version 29.0 for Macintosh) was used for all statistical tests, and GraphPad Prism software (version 9.3.1 for Macintosh) was used to create all graphs.

**Supplemental Results**

**Cocaine experience does not alter calnexin expression within vmPFC subregions.** To ensure that calnexin levels were unaffected by the cocaine history of the rats, the raw values for the calnexin immunoblots were compared between cocaine-naïve controls and cocaine-experienced rats tested for cue-elicited craving on WD3 or WD30. No group differences in calnexin expression was detected within the PL or IL of female rats [for PL, Withdrawal effect: F(3, 47)=1.044, p=0.313; Group effect: F(3,47)=0.966, p=0.331; interaction: F(3,47)=0.088, p=0.761; for IL, Withdrawal effect: F(3, 47)=0.500, p=0.483; Group effect: F(3,47)=0.234, p=0.631; interaction: F(3,47)=0.118, p=0.733]. Similarly, calnexin levels were unchanged by cocaine or withdrawal within the vmPFC of male rats [for PL: Withdrawal effect: F(3, 39)=1.008 p=0.322; Group effect: F(3,39)=0.094, p=0.331; interaction: F(3,39)=0.366, p=0.549; for IL, Withdrawal effect: F(3, 44)=0.118, p=0.732; Group effect: F(3,44)=0.206, p=0.653; interaction: F(3,44)=0.450, p=0.506]. See Figure 1 for representative immunoblots.

**Influence of estrous phase on the expression of NMDAR subunits and CaMKII in incubated cocaine-craving.** Previous research demonstrates that the magnitude of incubated cocaine-craving can be modulated by estrous phase [5-7] and within these same rats, we have shown that estrus females demonstrate higher responding on the active and inactive lever, than those in other phases of the estrous cycle [1; see **Suppl. Fig.1A**]. Within the present study, we found that this estrus effect on cocaine-seeking behavior was not associated with changes in GluN1, GluN2A, or GluN2B expression within the PL (**Suppl. Fig.1B-D**) [GluN1: F(1,27)<0.387; p>0.684, GluN2A:  F(1,27)<0.293, p>0.749; GluN2B: F(1,27)<0.384 p>0.685]. Similarly, we detected no changes associated with estrous phase in the expression of GluN1, GluN2A, or GluN2B  within the IL (**Suppl. Fig.1H-J**) [GluN1: F(1,27)<0.646; p>0.534, GluN2A:  F(1,27)<0.164, p>0.690; GluN2B: F(1,27)<0.242, p>0.787]. Within the PL, there was no effect of estrous phase on CaMKII, p(Thr286)-CaMKII, or relative CaMKII (**Suppl. Fig.1E-G**) [CaMKII: F(1,27)<0.184; p>0.834, p(Thr286)-CaMKII:  F(1,27)<0.122, p>0.730; CaMKII ratio: F(1,27)<0.007, p>0.993]. Finally, within the IL, we detected no effect of estrous phase on CaMKII (**Suppl. Fig.1K-L**) [F(1,27)<0.007, p>0.993] or p(Thr286)-CaMKII [F(1,27)<0.168, p>0.846]. There was a significant Withdrawal x Stage effect on relative p(Thr286)-CaMKII expression (**Suppl. Fig.1M**) [F(1,27)<5.504, p=0.012] and deconstruction along the Stage factor revealed a time-dependent increase in the relative p(Thr286)-CaMKII in estrus females [estrus: t(5)=2.525, p=0.053; diestrus: t(13)=1.602, p=0.133; proestrus: t(4)=1.416, p=0.230].

**Supplemental References**

1. Huerta Sanchez, L. L., Tadros, M. G., Doan, H. H. T., Vo, S. V., Chaudhari, S. R., Li, T. L., James, P. B., Na, A. Y., Cano, F. J., Kippin, T. E., & Szumlinski, K. K. (2025). AMPA/kainate receptor activation within the prelimbic cortex is necessary for incubated cocaine-craving. *Frontiers in psychiatry*, *16*, 1627477. <https://doi.org/10.3389/fpsyt.2025.1627477>
2. Cano, F. J., Denning, C. J. E., Udayashankar, H., Adler, S. D., Smith, K. E., Dang, V., Mohammadi, K., Reed Aparicio, A. T., Jotwani, A., Huerta Sanchez, L. L., & Szumlinski, K. K. (2025). Biomolecular correlates of incubated sucrose-seeking within ventromedial prefrontal cortex are sex- and subregion-selective. *Pharmacology, biochemistry, and behavior*, *256*, 174088. <https://doi.org/10.1016/j.pbb.2025.174088>
3. Miller, B.W., Wroten, M.G., Sacramento, A.D., Silva, H.E., Shin, C.B., Vieira, P.A., Ben‐Shahar, O., Kippin, T.E., & Szumlinski, K.K. (2016). Cocaine craving during protracted withdrawal requires PKCε priming within vmPFC. *Addiction Biology, 22*(3), 629–639. https://doi.org/10.1111/adb.12354
4. Szumlinski, K.K., Ary, A.W., Shin, C.B., Wroten, M.G., Courson, J., Miller, B.W., Ruppert-Majer, M., Hiller, J.W., Shahin, J.R., Ben-Shahar, O., Kippin, T.E. (2019) PI3K activation within ventromedial prefrontal cortex regulates the expression of drug-seeking in two rodent species. *Addiction Biology*, 24(6), 1216-1226. doi: 10.1111/adb.12696.
5. Corbett, C. M., Dunn, E., & Loweth, J. A. (2021). Effects of Sex and Estrous Cycle on the Time Course of Incubation of Cue-Induced Craving following Extended-Access Cocaine Self-Administration. *eNeuro*, *8*(4), ENEURO.0054-21.2021. <https://doi.org/10.1523/ENEURO.0054-21.2021>
6. Kerstetter, K. A., Aguilar, V. R., Parrish, A. B., & Kippin, T. E. (2008). Protracted time-dependent increases in cocaine-seeking behavior during cocaine withdrawal in female relative to male rats. *Psychopharmacology*, *198*(1), 63–75. <https://doi.org/10.1007/s00213-008-1089-8\>
7. Nicolas, C., Russell, T. I., Pierce, A. F., Maldera, S., Holley, A., You, Z. B., McCarthy, M. M., Shaham, Y., & Ikemoto, S. (2019). Incubation of Cocaine Craving After Intermittent-Access Self-administration: Sex Differences and Estrous Cycle. *Biological psychiatry*, *85*(11), 915–924. <https://doi.org/10.1016/j.biopsych.2019.01.015>

**Suppl. Table 1:  Means ± SEMs of the number of active and inactive lever-presses, as well as reinforcers earned, by male and female rats over the last 3 days of the cocaine or sucrose self-administration phases of the different experiments in this report.**

**Supplemental Figure Legends**

**Supplemental Figure 1:  Immunoblotting in mPFC subregions of cocaine-incubated female rats across the estrous cycle.** Summary of how the number of active and inactive lever-presses varied as a function of estrous cycle **(A)** (P=proestrus; E=estrus; D=diestrus; no females were found to be in metestrus). No relationship was observed between estrous cycle phase and the expression of NMDAR subunits within the PL **(B-D)** or CaMKII activation in this subregion **(E-G)**. No estrous cycle effects were detected for the IL expression of NMDAR subunits **(H-J)**, although estrus females exhibited a time-dependent increase in the relative expression of p(Thr286)-CaMKII **(K-M)**. The data represent the means ± SEMs of the individual animals indicated. +p<0.05 WD3 vs. WD30 (incubation).

**Suppl. Figure 1**
